# Neurometabolic and structural alterations of medial septum and hippocampal CA1 in a model of post-operative sleep fragmentation in aged mice: a study combining 1H-MRS and DTI

**DOI:** 10.3389/fncel.2023.1160761

**Published:** 2023-06-02

**Authors:** Yun Li, Lina Zhao, Kai Zhang, Mengxi Shen, Yize Li, Yang Yu, Jiafeng Yu, Jingyu Feng, Keliang Xie, Yonghao Yu

**Affiliations:** ^1^Department of Anesthesiology, Tianjin Medical University General Hospital, Tianjin, China; ^2^Tianjin Research Institute of Anesthesiology, Tianjin, China; ^3^Department of Critical Care Medicine, Tianjin Medical University General Hospital, Tianjin, China

**Keywords:** post-operative sleep disturbance, sleep fragmentation, 1H-MRS, DTI, aging, medial septum, hippocampus, CA1

## Abstract

Post-operative sleep disturbance is a common feature of elderly surgical patients, and sleep fragmentation (SF) is closely related to post-operative cognitive dysfunction (POCD). SF is characterized by sleep interruption, increased number of awakenings and sleep structure destruction, similar to obstructive sleep apnea (OSA). Research shows that sleep interruption can change neurotransmitter metabolism and structural connectivity in sleep and cognitive brain regions, of which the medial septum and hippocampal CA1 are key brain regions connecting sleep and cognitive processes. Proton magnetic resonance spectroscopy (1H-MRS) is a non-invasive method for the evaluation of neurometabolic abnormalities. Diffusion tensor imaging (DTI) realizes the observation of structural integrity and connectivity of brain regions of interest *in vivo*. However, it is unclear whether post-operative SF induces harmful changes in neurotransmitters and structures of the key brain regions and their contribution to POCD. In this study, we evaluated the effects of post-operative SF on neurotransmitter metabolism and structural integrity of medial septum and hippocampal CA1 in aged C57BL/6J male mice. The animals received a 24-h SF procedure after isoflurane anesthesia and right carotid artery exposure surgery. 1H-MRS results showed after post-operative SF, the glutamate (Glu)/creatine (Cr) and glutamate + glutamine (Glx)/Cr ratios increased in the medial septum and hippocampal CA1, while the NAA/Cr ratio decreased in the hippocampal CA1. DTI results showed post-operative SF decreased the fractional anisotropy (FA) of white matter fibers in the hippocampal CA1, while the medial septum was not affected. Moreover, post-operative SF aggravated subsequent Y-maze and novel object recognition performances accompanied by abnormal enhancement of glutamatergic metabolism signal. This study suggests that 24-h SF induces hyperglutamate metabolism level and microstructural connectivity damage in sleep and cognitive brain regions in aged mice, which may be involved in the pathophysiological process of POCD.

## 1. Introduction

Sleep is an essential physiological phenomenon for human beings (Maquet, 2001). Good sleep after anesthesia and surgery not only promotes physical recovery, but also is essential for maintaining brain health, especially in elderly patients (O’Gara et al., 2021). However, due to various factors such as environment, medical treatment and patients, more than 40% of elderly surgical patients have been unable to keep normal sleep quality in the post-operative stage and suffer from sleep disorders, especially sleep fragmentation (SF) (Lin et al., 2021; Stewart and Arora, 2022). SF does not shorten the total sleep time, but its impact on sleep is mainly manifested as the interruption of sleep continuity caused by repeated awakenings, thereby destroying the overall structure of sleep, similar to obstructive sleep apnea (OSA) (Raiesdana, 2017). Post-operative SF is associated with postoperative cognitive dysfunction (POCD) (Lu et al., 2020), but its underlying neuroinflammatory mechanism has not fully elucidated this association (Vacas et al., 2017). Studies have shown that the process of cognitive impairment induced by sleep disorders is often accompanied by neurotransmitter metabolism disorder and structural integrity damage in medial septum and hippocampus, key brain regions for sleep and cognition (Puskar et al., 2021; Leung and Ma, 2022). However, the neurometabolic mechanism of post-operative SF has not been elucidated, which also hinders the intervention of POCD in elderly surgical patients.

Previous studies on post-operative sleep disorders have focused on electroencephalogram and behavior (Hillman, 2017; Lin et al., 2021), but few studies on neurotransmitter metabolism and functional brain structure. Many studies have shown that sleep deprivation can lead to structural changes in the hippocampus, including decreased density of dendritic spine and reduced neurogenesis (Kreutzmann et al., 2015; Gisabella et al., 2020). Chronic sleep deprivation may even lead to decreased hippocampal volume, which is involved in cognitive impairment (Noh et al., 2012). Neuroimaging techniques provide a means of visualizing metabolic and structural changes in functional brain regions at high resolution *in vivo*. Proton magnetic resonance spectroscopy (1H-MRS) is the only imaging technique for non-invasive detection of brain tissue and biochemical metabolism *in vivo* and are often earlier than the changes of conventional MR structural imaging to detect abnormalities. 1H-MRS has been shown to be an important *in vivo* tool for studying the neurometabolic mechanisms of sleep and cognitive diseases, involving glutamatergic metabolic signals, gamma-aminobutyric acid (GABA), acetylcholine, and N-acetylaspartate (NAA) (Guan et al., 2022). Diffusion tensor imaging (DTI), an imaging method to detect the diffusion motion of water molecules *in vivo*, has made the non-invasive study of anatomical connectivity a reality. Studies have shown that sleep disorders damage the integrity of white matter fibers in brain regions related to sleep and cognition (Raikes et al., 2018; Grumbach et al., 2020), indicating that the impaired anatomical connectivity between brain regions may be the morphological basis of neurocognitive disorders induced by sleep disorders. However, little is known about the effects of post-operative SF on brain neuro-metabolism and microstructure in elderly patients.

Mainstream studies suggest that the changes of sleep and sleep associated cognitive function in aged animal models are closely related to the synergistic disorder of brain regions of medial septum (MS) and hippocampus associated with sleep and cognitive. MS has a two-way connection with hippocampal CA1 region, which is the key hippocampal subregion to determine cognition (Muller and Remy, 2018). The emergence of magnetic resonance imaging (MRI) involving changes in neurotransmitters and structural connections has become a powerful technique for neuroimaging of brain activity stimulation *in vivo*, including SF and cognitive impairment, and provides a new insight for studying the neuroimaging changes and mechanisms of the potentially key brain regions MS and hippocampal CA1 in post-operative SF. In this study, the information of brain regional changes in post-operative SF and their correlation with POCD were investigated through a combination of behavioral, 1H-MRS and DTI methods in aged mice.

## 2. Materials and methods

### 2.1. Animals

**FIGURE 1:**
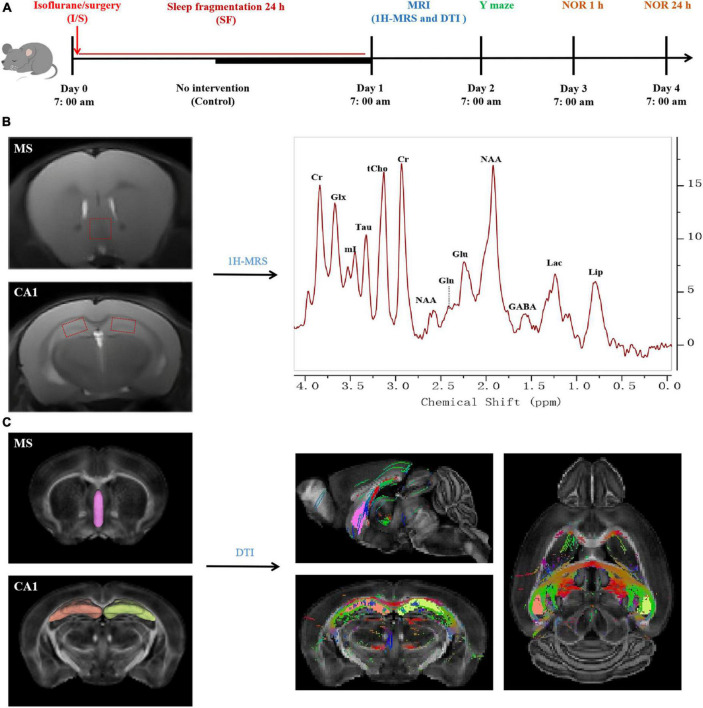
Flow chart of the procedures and magnetic resonance imaging (MRI) processing in the study. **(A)** Experimental protocol. Aged C57BL/6J mice were divided into three groups: Control, isoflurane and surgery (I/S), and I/S + sleep fragmentation (I/S + SF) groups. Group I/S + SF received 24-h sleep fragmentation following isoflurane anesthesia and right carotid artery exposure surgery, group I/S received isoflurane anesthesia and right carotid artery exposure surgery, and group Control received no intervention. MRI study of diffusion tensor imaging (DTI) and proton magnetic resonance spectroscopy (1H-MRS), and behavior tests of Y maze, 1 h and 24 h novel object recognition (NOR) were performed. **(B)** 1H-MRS data processing and analysis. The volumes of interest were set at medial septum (MS) and CA1. Typical spectra were recorded to analyze the changes of neurometabolic signals. Glu, glutamate; Gln, glutamine; Glx, glutamate/glutamine; GABA, gamma-aminobutyric acid; tCho, total choline; mI, myo-Inositol; Tau, taurine; NAA, N-acetylaspartate; Cr, creatine; Lac, lactate; Lip, lipid. **(C)** DTI data processing and analysis (reprinted from DSI studio). MS and CA1 were identified as regions of interest. DTI signal intensity of the regions of interest were subjected to structural integrity analysis.

Male C57BL/6J mice aged 18 months were housed in the same at constant temperature (25 ± 2°C), humidity (40 ± 10%) and 12/12-h light-dark cycle (lights on at 7 a.m. and lights off at 7 p.m.). The mice were allowed to eat and drink freely and adapt to the test environment for at least 1 week. The mice were randomly allocated to three groups: mice were subjected to no intervention (Control), mice were anesthetized with isoflurane and subjected to a right carotid artery exposure surgery (I/S), I/S mice were subjected to a 24-h sleep fragmentation procedure (I/S + SF). MRI and behavioral tests were subsequently performed. Flow chart of the animal experiments is shown in Figure 1. All the experiments followed the national animal experiment management standards and was approved by the Animal Ethics Committee of the General Hospital of Tianjin Medical University.

### 2.2. Anesthesia and surgery

Mice were subjected to the right carotid artery exposure surgery under isoflurane anesthesia (Zheng et al., 2017). The animals were placed in a small animal anesthesia box with carrier gas of 100% O_2_ at 1 L/min and were induced by inhalation of 3.0% isoflurane for 5 min. After induction, 1.5% isoflurane was administered through nasal mask to maintain anesthesia. Five min after induction of anesthesia, the eye ointment was applied to both eyes and 2 ml of warm sterile saline was injected subcutaneously. After induction of general anesthesia, the mice were fixed in the supine position on the surgical plate, and about 1.5 cm incision was made in the midline of the neck. The soft tissue on the trachea was gently separated. 1 cm long right common carotid artery was carefully dissected from the adjacent tissue without damaging the vagus. The wound was then irrigated and closed with surgical sutures, and local injections of 2% lidocaine were used to treat post-operative pain. The procedure was performed under sterile conditions and the operation time was about 20 min. The mice were resuscitated on dry and warm bedding and returned to the cage after awakening.

### 2.3. Sleep fragmentation

Post-operative SF modeling was established with a validated sleep-interruption device at 7 a.m., which used an automated scanning rod to interrupt sleep by haptically stimulating the mice to move around the cage (Sinton et al., 2009). The cycle time was set to 2 min (30-s on/90-s off), and the deprivation rod was scanned cycled at a fixed rate (100 r/min) to achieve 30 sleep interruptions/h. The cage arrangement allowed the mice to eat and drink freely during the modeling period. This method of sleep disruption has been reported to produce moderate to severe sleep fragmentation without causing total sleep deprivation, preserving sleep macroscopic or microscopic structures of SF. Post-operative SF procedure lasted for 24 h.

### 2.4. Magnetic resonance imaging

*In vivo* MRI were conducted with a 9.4T magnetic resonance small animal scanner (Bruker Biospin, Germany) at the Multimodal Clinical Pre-molecular Imaging Center, Tianjin Medical University General Hospital. Mice were induced with 3% isoflurane, their heads were fixed in magnets using a bite rod and blunt earplugs, and maintained with 1−1.5% isoflurane. The respiratory rate and body temperature of mice were monitored using small animal monitoring system. The temperature of the mice was maintained at 36−37°C using warm water circulation.

Proton magnetic resonance spectroscopy images were collected on the volumes of interest (VOIs) of medial septum (MS, 2.0 × 2.0 × 2.0 mm^3^) and Hippocampal CA1 region (CA1, 1.5 × 2.0 × 1.5 mm^3^) using a Point-RESolved Spectroscopy (PRESS) sequence: TR = 2,500 ms, TE = 15 ms, and number of averages = 256. Before scanning, field shimming was implemented, and the water signal was suppressed. Individual spectra were obtained without suppressing the water signal as a reference for determining the corresponding metabolite concentration. 1H-MRS images were analyzed with linear combination of model (LCModel) software. The levels of glutamate (Glu), glutamine (Gln), glutamate/glutamine (Glx), GABA, total choline (tCho), myo-Inositol (mI), taurine (Tau), NAA, and creatine (Cr) in the VOIs were estimated by the LCModel method (Provencher, 2001).

Diffusion tensor imaging images were obtained by a spin-echo echo-planar imaging (EPI) sequence: TR = 3,000 ms, TE = 19.6 ms, gradient directions = 85, diffusion gradient on time = 4 ms, field of view (FOV) = 20 × 20 mm^2^, slice thickness = 0.5 mm, and matrix = 256 × 256. After the acquisition of 5 images acquired with *b* = 0 s/mm^2^, two b values of 2,000 s/mm^2^ and 4,000 s/mm^2^ were acquired for each direction. DTI images were processed and analyzed using ParaVision 360 V3.0 software (Bruker Biospin, Germany) and DSI studio.^1^ The ROIs were the same as 1H-MRS. The parameters of DTI on the ROIs including fractional anisotropy (FA), mean diffusivity (MD), axial diffusivity (AD), and radial diffusivity (RD) were estimated with the DSI studio automatic program (Liu et al., 2021).

For the analysis of hippocampal structure volume, T2-weighted images were obtained in coronal plane using a 3D rapid acquisition with relaxation enhancement (RARE) sequence: TR = 7,750 ms, TE = 33 ms, FOV = 20 × 20 mm^2^, slice thickness = 0.5 mm, matrix = 256 × 256, and number of segments = 4. For volume analysis, ITK-SNAP software was used to manually track the hippocampus on a continuous T2-weighted 3D image slice containing the entire hippocampus (Yushkevich et al., 2006). Hippocampal segmentation was performed in the coronal plane view. The boundary of hippocampus was determined by referring to the stereotactic map of mouse brain, and the volume of hippocampus was calculated according to the number of segmented voxels (Brait et al., 2021).

### 2.5. Behavioral tests

#### 2.5.1. Y-maze test

Y-maze test was used to detect the short-term spatial memory ability of mice (Kraeuter et al., 2019). The device was composed of three arms (30 cm × 8 cm × 20 cm) made of medical organic board, with an Angle of 120° and a movable partition at the central junction. The three arms of the maze were randomly set as: in the first stage of training, the mice were gently placed in the three-equal-arm maze for adaptive training for 10 min to adapt to the environment, and the new arm was closed at this time. The mice were put in by the starting arm and allowed to explore freely in the maze for 5 min. The ANY-maze video monitoring and analysis system (Stoelting Company, CO, USA) was used to record the total exploration time, distance and entry times in each arm. Clean the test chamber after each test and wipe the chamber with 75% alcohol. The longer the mice spent exploring the novel arm, the stronger their spatial memory.

#### 2.5.2. Novel object recognition test

Novel object recognition (NOR) was used to test the recognition memory ability of mice (Snigdha et al., 2014). Mice were gently placed in an empty box (40 cm × 40 cm × 50 cm) made of medical organic plates for 10 min to adapt to the environment. In the training stage, two identical objects were placed symmetrically in the box, and mice were placed into the box with their back facing, with the same distance from the tip of their nose to the two objects. The mice were allowed to explore the two objects freely for 5 min, and the total exploration time of the two objects was recorded by the ANY-Maze video monitoring and analysis system (Stoelting Company, CO, USA). After 1 h and 24 h, one object in the test box was replaced by another object with different shape and color, with the same position. The mice were put into the test box again and allowed to explore freely for 5 min, and the exploration time of the novel object was recorded. Clean the test chamber after each test and wipe the object and chamber with 75% alcohol. The discrimination index [novel object exploration time ÷ (novel object exploration time + old object exploration time) × 100%] was calculated to evaluate the learning and memory ability of mice.

### 2.6. Statistical analysis

Statistical analyses were performed using GraphPad Prism 9.4. Measurement data were described as mean ± SME. ANOVA was used for multi-group analyses. Tukey Kramer multiple comparison test was used for pairwise comparison between two groups. *P* < 0.05 was considered as statistically significant.

## 3. Results

### 3.1. Changes in the MS and hippocampal CA1 region at the neurometabolic level in response to post-operative sleep fragmentation

**FIGURE 2:**
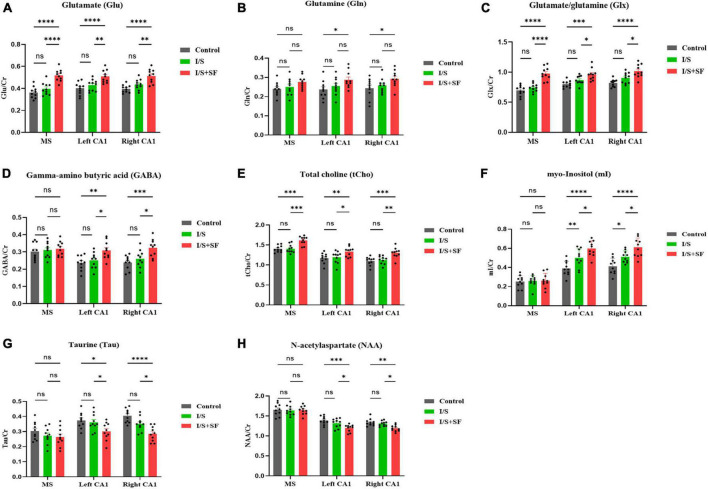
Comparison of 1H-MRS data in MS and CA1 regions in the three groups. Column charts of glutamate (Glu) **(A)**, glutamine (Gln) **(B)**, glutamate/glutamine (Glx) **(C)**, gamma-aminobutyric acid (GABA) **(D)**, total choline (tCho) **(E)**, myo-Inositol (mI) **(F)**, taurine (Tau) **(G)**, and N-acetylaspartate (NAA) **(H)** show alterations of the concentration ratio of the neuro-metabolites to creatine (Cr). Data are expressed as mean ± SME (*n* = 10/group). Symbols representing significant differences in the ANOVA of the Control, I/S and I/S + SF groups. ^ns^*P* > 0.05; *P < 0.05; ^**^P < 0.01; ^***^P < 0.001; ^****^P < 0.0001.

An *in vivo* 1H-MRS at 9.4T was performed to assess neurometabolic changes in the MS and hippocampal CA1 region in the Control, I/S and I/S + SF groups. Lactate (Lac) and lipid (Lip) were not detected in the MS and CA1 in the three groups. 1H-MRS results showed alterations in neurometabolite levels immediately after post-operative 24-h SF exposure (Figure 2). Post-operative SF significantly increased the ratios of glutamatergic metabolites Glu (*F* = 45.67, *P* < 0.0001, Figure 2A) and Glx (*F* = 48.99, *P* = 0.0001, Figure 2C) to Cr in the MS, left and right CA1, but the change of Gln/Cr was not significant (*F* = 0.3837, *P* = 0.6826, Figure 2B) in the MS. Post-operative SF significantly increased the ratio of Gln/Cr (*F* = 8.679, *P* = 0.0004) in bilateral hippocampus compared with the Control but not the I/S. Isoflurane anesthesia and surgery did not significantly alter the ratios of Glu/Cr, Gln/Cr, and Glx compared with the Control (*P* > 0.05). The ratios of GABA/Cr (*F* = 7.797, *P* = 0.0008) in the left and right CA1 showed a significant increase in the I/S + SF compared with the Control and I/S, but the change in the MS was not significant in the three groups (*F* = 1.682, *P* = 0.1621, Figure 2D). I/S increased the ratio of GABA/Cr in the left and right CA1 compared with the Control, but the change was not significant. I/S + SF showed a significant increase in the ratio of tCho/Cr (*F* = 25.41, *P* < 0.0001, Figure 2E) in the MS, left and right CA1 compared with the Control and I/S. There was no significant difference in the ratios of tCho/Cr (*F* = 0.2336, *P* = 0.9187) in the MS, left and right CA1 between the Control and I/S (*P* > 0.05). Compared with the Control and I/S, the ratio of mI/Cr (*F* = 23.76, *P* = 0.0001, Figure 2F) in the left and right CA1 in the I/S + SF increased significantly. The ratio of mI/Cr in the MS showed no significant difference in the three groups (*P* > 0.05). The ratios of Tau/Cr (*F* = 15.11, *P* < 0.0001, Figure 2G), and NAA/Cr (*F* = 9.792, *P* = 0.0002, Figure 2H) in the left and right CA1 showed a significant decrease in the I/S + SF compared with the Control and I/S, but the change in the MS was not significant in the three groups (*P* > 0.05). There was no significant difference in the ratios of Tau/Cr (*F* = 1.698, *P* = 0.1585) and NAA/Cr (*F* = 2.208, *P* = 0.0754) in the left and right CA1 between the Control and I/S.

### 3.2. Changes in structural integrity of the MS and hippocampal CA1 region following post-operative sleep fragmentation

**FIGURE 3:**
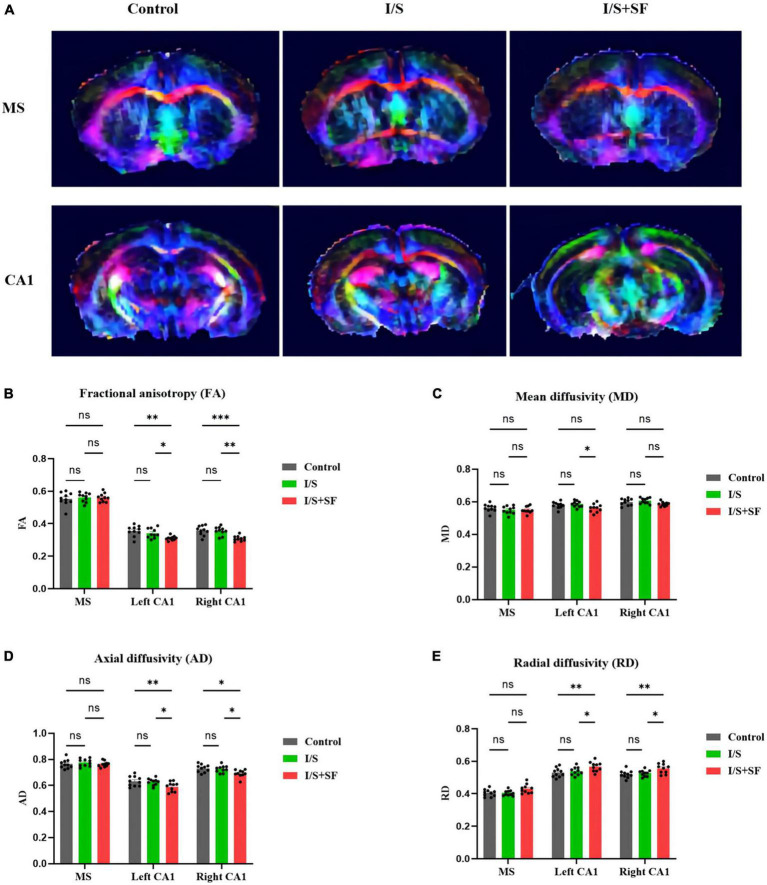
Comparison of DTI data in MS and CA1 regions in the three groups. **(A)** Representative images of DTI in the MS and CA1 regions of mice in the Control, I/S and I/S + SF groups. **(B)** Quantitative analysis of fractional anisotropy (FA) in the Control, I/S and I/S + SF groups. **(C)** Quantitative analysis of mean diffusivity (MD) in the Control, I/S and I/S + SF groups. **(D)** Quantitative analysis of axial diffusivity (AD) in the Control, I/S, and I/S + SF groups. **(E)** Quantitative analysis of radial diffusivity (RD) in the Control, I/S and I/S + SF groups. Data are expressed as mean ± SME (*n* = 10/group). Symbols representing significant differences in the ANOVA of the Control, I/S and I/S + SF groups. ^ns^*P* > 0.05; *P < 0.05; ^**^P < 0.01; ^***^P < 0.001.

*In vivo* DTI study showed that post-operative SF impaired the integrity and connectivity of white matter fibers in the MS and hippocampal CA1 region (Figure 3). DTI showed color maps of representative white matter fiber tracts in the MS and hippocampal CA1 region in the three groups (Figure 3A). The I/S + SF showed significantly lower FA values in the left and right CA1 than the Control and I/S (*F* = 3.533, *P* = 0.0104, Figure 3B). Isoflurane anesthesia and surgery did not significantly change FA values compared with the Control (*P* > 0.05). Post-operative SF only significantly changed the MD values in the left CA1 compared with the I/S (*F* = 5.052, *P* = 0.0086, Figure 3C), and the changes was not significant in the right CA1. The AD values in the left and right CA1 were significantly lower in the I/S + SF than that in the Control and I/S (*F* = 5.052, *P* = 0.0086, Figure 3D). There was no significant difference in the AD values in the left and right CA1 between the Control and I/S groups (*P* > 0.05). In contrast, the I/S + SF showed significantly higher RD values in the left and right CA1 than the Control and I/S (*F* = 14.35, *P* < 0.0001, Figure 3E). The I/S showed no significant difference compared with the Control (*P* > 0.05). DTI metrics measurement and analysis of white matter tracts in the MS showed no significant difference in the mean FA and values in the Control, I/S and I/S + SF groups (*P* > 0.05).

### 3.3. Measurement and analysis of hippocampal volume in post-operative sleep fragmentation model

**FIGURE 4:**
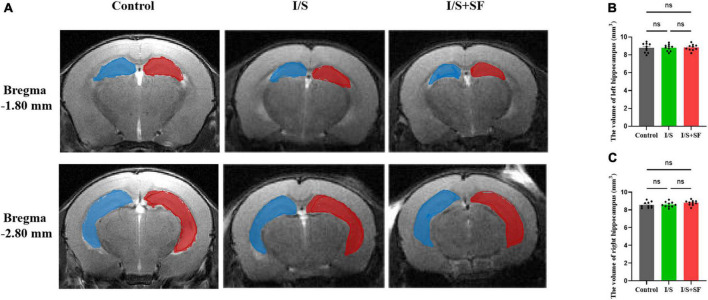
Hippocampal volume. **(A)** Representative T2-weighted images of the left (blue) and right (red) hippocampus in the Control, I/S and I/S + SF groups. **(B)** Quantitative analysis of the volume of left hippocampus in in the Control, I/S and I/S + SF groups. **(C)** Quantitative analysis of the volume of left hippocampus in in the Control, I/S and I/S + SF groups. Data are expressed as mean ± SME (*n* = 10/group). ns representing no significant difference with *P* > 0.05 in the ANOVA of the Control, I/S and I/S + SF groups.

T2-weighted MRI showed no significant changes in the hippocampal volume in mice with post-operative SF (Figure 4). The representative T2-weighted and 3D images of the hippocampus in the three groups showed the artificial segmentation of the left (blue) and right (red) hippocampus (Figure 4A). No significant change in the left and right hippocampal volume (*F* = 1.580, *P* = 0.2244, Figure 4B; *F* = 1.493, *P* = 0.2426, Figure 4C) were observed in the Control, I/S and I/S + SF groups (*P* > 0.05).

### 3.4. Effects of post-operative sleep fragmentation on cognitive function

**FIGURE 5:**
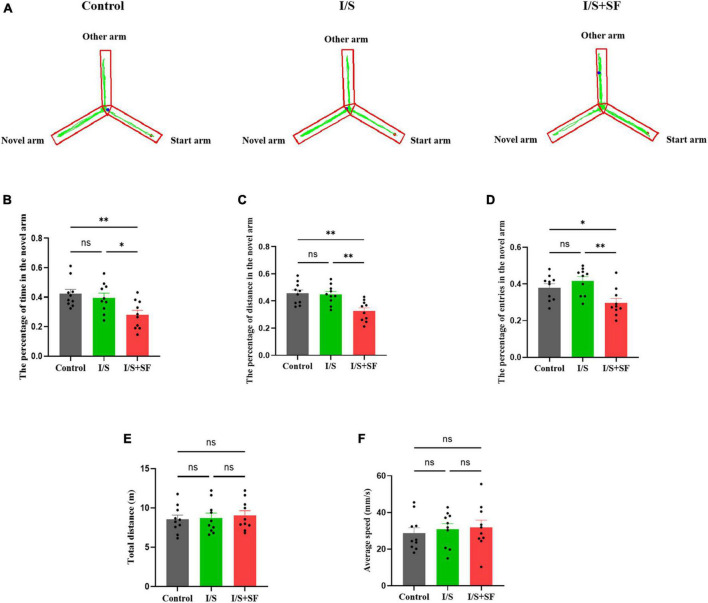
Short-term spatial memory in the Y maze test. **(A)** Representative trajectories of mice in the Y maze in the Control, I/S and I/S + SF groups. **(B–D)** Quantitative analysis of the percentage of time, distance and entries in the novel arm in the Control, I/S and I/S + SF groups. **(E)** Quantitative analysis of total distance in the Control, I/S and I/S + SF groups. **(F)** Quantitative analysis of average speed in the Control, I/S and I/S + SF groups. Data are expressed as mean ± SME (*n* = 10/group). Symbols representing significant differences in the ANOVA of the Control, I/S and I/S + SF groups. ^ns^*P* > 0.05; *P < 0.05; ^**^P < 0.01.

In the Y-maze test, post-operative 24-h SF induced subsequent short-term memory impairment (Figure 5). Representative trajectories of mice in the three groups were shown in Figure 5A. A significantly lower percentage of time (*F* = 6.159, *P* = 0.0063, Figure 5B), distance (*F* = 9.280, *P* = 0.0009, Figure 5C) and entries (*F* = 7.056, *P* = 0.0034, Figure 5D) in the novel arm, indicative of spatial working memory deficit, was observed in the I/S + SF group, compared with the Control and I/S groups. The mice receiving post-operative 24-h SF performed significantly worse than the Control and I/S mice. The I/S group showed similar the percentage of time, distance and entries in the novel arm as the Control (*P* > 0.05), indicating no spatial memory impairment. No significant differences in the total distance (*F* = 0.1851, *P* = 0.8320, Figure 5E) and average speed (*F* = 0.2589, *P* = 0.7738, Figure 5F) were detected in the Control, I/S and I/S + SF groups (*P* > 0.05), showing similar locomotor activity in the mice.

**FIGURE 6:**
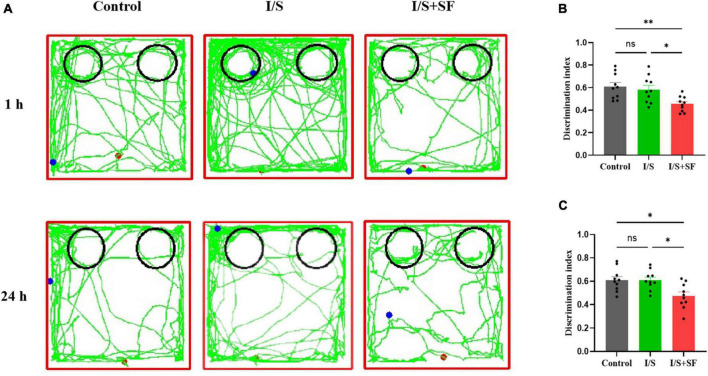
Short- and long-term recognition memory in the NOR test. **(A)** Representative trajectories of mice in the NOR in the Control, I/S and I/S + SF groups. **(B)** Quantitative analysis of discrimination index in 1-h NOR in the Control, I/S and I/S + SF groups. **(C)** Quantitative analysis of discrimination index in 24-h NOR in the Control, I/S and I/S + SF groups. Data are expressed as mean ± SME (*n* = 10/group). Symbols representing significant differences in the ANOVA of the Control, I/S and I/S + SF groups. ^ns^*P* > 0.05; *P < 0.05; ^**^P < 0.01.

In the NOR test, post-operative 24-h SF impaired short-term and long-term recognition memory (Figure 6). As shown Figure 6A, representative trajectories of 1-h and 24-h NOR of mice in the three groups were displayed. The 1-h NOR test showed a significantly lower discrimination index in the I/S + SF than that in the Control and I/S (*F* = 6.512, *P* = 0.0049, Figure 6B), but no significant difference in the discrimination index between the Control and I/S groups (*P* > 0.05). Similarly, the I/S + SF exhibited a higher discrimination index in the 24-h NOR test, compared with the Control and I/S groups (*F* = 6.660, *P* = 0.0045, Figure 6C), and no significant difference in discrimination index in the 24-h NOR test were detected between the Control and I/S groups (*P* > 0.05). Taken together, these data demonstrated post-operative 24-h SF in mice worsened their short-term (1 h) and long-term (24 h) recognition memory. However, isoflurane anesthesia and surgery showed no impaired cognitive function, similar to the Control mice.

## 4. Discussion

Sleep disturbance leads to neurotransmitter disorder and white matter damage, which is one of the main reasons for age-related cognitive impairment as well as POCD. In perioperative sleep disturbance models, the post-operative SF model induces a strong neuroinflammatory response and simulates a characteristic pathogenesis of POCD (Lu et al., 2020), but cannot fully explain the underlying neuropathological process (Vacas et al., 2017). Research has shown that glutamatergic, GABA, and cholinergic projections were in the MS-CA1 (Muller and Remy, 2018). The first manifestation of their dysfunction is neurotransmitter disorder, which induces brain dysfunction, including sleep disorders, Alzheimer’s disease, dementia, and so on (Chen et al., 2021). In addition, the integrity and connectivity of white matter fiber tracts are also the key structural basis that determines the function of sleep and cognition brain regions. In our study, we detected that post-operative SF significantly evoked substantial neurometabolic activation in the MS and hippocampal CA1 region, especially glutamatergic metabolic signals, accompanied by demyelination of white matter fiber tracts and microstructure damage in hippocampal CA1 region. Importantly, post-operative SF with neurotransmitter disorder and impaired structural integrity significantly impaired subsequent hippocampus-dependent cognitive behavior. This study revealed that SF induced major neurotransmitter disturbance and structural integrity damage in the key brain areas of MS and hippocampal CA1, which may be a potential new mechanism of POCD induced by SF in the elderly.

Existing evidence supports the important role of MS-hippocampal CA1 neural connections in sleep and cognitive function (Qureshi and Jha, 2017; Muller and Remy, 2018; Delorme et al., 2021). MS is considered to be a key brain region regulating sleep-wake state and hippocampus-dependent memory, and has become a research hotspot in the field of sleep cognition in recent years. MS contains highly interconnected neurons: cholinergic, gamma-aminobutyric acid (GABA), and glutamatergic neuronal populations synchronously coordinate sleep-wake and cognitive processes (Griguoli and Pimpinella, 2022). The hippocampal CA1 region is considered to be the core region of hippocampus-dependent learning and memory processing, and it mainly forms interconnections with the cerebral cortex (Uebele et al., 2009), which is the key region to determine spatial learning and memory (Yousefi et al., 2012). Our study confirms that the MS and hippocampal CA1, as well as neurometabolic and microstructural changes in these brain regions, are involved in the development of sleep fragmentation-induced POCD in aged mice. Notably, sleep disorders, including SF, result in functional and structural changes in multiple brain regions. Interestingly, preoptic-anterior hypothalamus (POAH), a major integrator of sleep and body temperature regulation information, is also a hot spot. POAH integrates information on alertness state, body temperature, and ambient temperature, and affects alertness state and body temperature. Studies have shown that GABA in the medial POAH region induces sleep through GABA-A receptors and prevents hyperthermia (Jha et al., 2001; Jha and Mallick, 2009), which provides A different perspective to explore the new role of sleep.

Proton magnetic resonance spectroscopy is a non-invasive technique to detect the neurometabolic pathophysiology of brain functional abnormalities prior to structural changes (Guan et al., 2022). 1H-MRS has been successfully applied to clinical study of brain metabolism mechanism of OSA (Yadav et al., 2014). Sleep fragmentation shares a similar feature of sleep disruption with OSA (Raiesdana, 2017). The relative increase or decrease in the levels of some neurotransmitters and amino acids, such as glutamate (Glu), glutamine (Gln), glutamate/glutamine (Glx), gamma-aminobutyric acid (GABA), total choline (tCho), myo-Inositol (mI), taurine (Tau), N-acetylaspartate (NAA), and creatine (Cr), provide important clues to the underlying neurometabolic mechanisms in key brain regions related to post-operative SF. To analyze the function of these metabolites, we used the ratio of spectral peaks of these metabolites to Cr, which was explained by the metabolic stability of Cr in non-traumatic and non-tumor brain tissues.

In this study, we observed strong changes in glutaminergic metabolism signals in the MS and hippocampal CA1 induced by post-operative SF. Glu plays a crucial role in supporting the sleep-wake cycle and cognitive processes (Allen et al., 2013; Kroll et al., 2018). Excessive extracellular Glu overstimulation of metabolism and ionic Glu receptors is a major culprit in neuronal excitatory toxicity and leads to neuropsychiatric disorders that can be exacerbated by sleep deprivation (Del Cid-Pellitero et al., 2017). Our data further revealed that sleep disruption also induced the hyperactivation of Glu and Glx in MS and hippocampal CA1 region, and resulted in the decrease of NAA/Cr in hippocampal CA1 region, suggesting the impairment of hippocampal neuronal excitability. Interestingly, the neuroanatomical study of MS showed that 23% of the neuronal types of MS projections to hippocampal CA1 were glutamatergic neurons which played a key role in exploring behavior and cognitive function in mice by regulating hippocampal theta rhythm (Muller and Remy, 2018). The 1H-MRS data of MS strongly suggest that sleep disruption does affect different levels of glutamatergic neuronal activation. More specifically, sleep fragmentation increased Glu and Glx metabolism levels in the MS region, and the changes were at low levels in the Control mice. Similarly, the metabolic ratio of Glu and Glx in the hippocampal CA1 region was significantly increased after sleep fragmentation, suggesting that MS-CA1 glutamatergic projections were activated at the metabolic level. In the study of the neural mechanism of sleep and cognition, the use of genetic tools to manipulate neural circuits is the latest research method, but they are complex, time-consuming and technically difficult to operate. However, they lack the ability to simultaneously present multiple neurotransmitters at the *in vivo* level. 1H-MRS is an available imaging tool for studying neural metabolism as an auxiliary method to explore the neural connectivity of brain areas in sleep cognition.

Basic studies *in vitro* and in animal models have highlighted the prominent role of cholinergic mechanism in regulating sleep and cognition (George et al., 2006). In the terminals of cholinergic neurons, choline and acetyl coenzyme A generate acetylcholine (ACh) under the action of choline acetyltransferase. The tCho of 1H-MRS reflects the total choline content in the brain, including choline phosphate, phosphatidyl choline and glycerine phosphate choline (Wang et al., 2008). The peak value of tCho is determined by the choline concentration in membrane phospholipid, which is usually related to the synthesis and catabolism of cell membrane, and also the precursor of the neurotransmitter ACh (Donovan et al., 2022). Recent studies have shown that tCho is significantly correlated with ACh in the hippocampus and can accurately reflect ACh changes in cholinergic neurons (Wang et al., 2008). In this study, our data confirmed that post-operative SF enhanced the level of tCho metabolism in the MS, accompanied by an also upregulation of tCho in the CA1 region. The results may imply that MS-CA1 cholinergic projections are activated by post-operative SF. Most of the cholinergic neurons in MS can project to various subregions of the hippocampus (Muller and Remy, 2018), especially cholinergic nerve fibers in the CA1 subregion, which has an important impact on hippocampal oscillation and synaptic plasticity and is considered to be related to the consolidation process of memory (McQuiston, 2010). One study has found that 6-h sleep loss can selectively activate cholinergic input signals to the hippocampus and activate the SST intermediate neurons that produce GABA, thus damaging the memory consolidation process (Delorme et al., 2021). Our study also observed the phenomenon of GABA metabolism enhancement induced by post-operative 24-h sleep fragmentation in hippocampus CA1 region. The cholinergic nervous system is a kind of neural network in the brain that is closely related to sleep and learning and memory functions (Yousefi et al., 2012). It has been found that ACh levels in the hippocampus can bi-directionally regulate cognitive processes in different animal models of sleep disorders, which is mainly related to the inhibition of GABAergic neural network generated by ACh feedback activation. Glutamatergic, cholinergic, and GABAergic neurons jointly control the sleep-wake process, and while prolonged wakefulness enhances the activity of excitatory neurons, it also activates inhibitory neurons to release GABA through glutamate and acetylcholine (Brown et al., 2012; Del Cid-Pellitero et al., 2017). This process is caused by the homeostatic regulation of the body’s spontaneous return to normal sleep-wake rhythm, but the enhanced neural projection of MS to the hippocampus can also pay the price of cognitive impairment.

Sleep fragmentation leads to aging-related cognitive decline, which may trigger neuroinflammatory responses through glial activation (Kaneshwaran et al., 2019). In the brain, mI is mainly synthesized in glial cells and cannot cross the blood-brain barrier (Plitman et al., 2016). Therefore, mI is traditionally considered as a marker of glial cells. Previous studies have demonstrated that mI is more abundant in glial cells than in neurons and can be detected by 1H-MRS (Ding and Lanfermann, 2015). Notably, glial disorders are highly correlated with the pathophysiology of sleep disorders (Wang et al., 2021). In this study, the result of mI in the hippocampus CA1 suggested that post-operative SF activated hippocampal glial activity. Interestingly, elevated MI-CHO levels are often interpreted as glial activation which is associated with neuroinflammatory responses. Our study also observed the increased levels of mI and tCho in the hippocampal CA1. Studies have shown that glial proliferation in the CA1 region of the hippocampus impairs neuronal integrity (Maquet, 2001; McQuiston, 2010). In addition, NAA has been proved to be an indicator of neuronal integrity in 1H-MRS (Kroll et al., 2018). Our results also confirmed that the increase of mI was accompanied by the decrease of NAA in CA1 region, which may imply that glial activation indirectly leads to the damage of CA1 neurons. Together, the data support the emerging hypothesis that the mI is a key player in post-operative SF-induced hippocampal neuronal damage.

Diffusion tensor imaging is a non-invasive *in vivo* technique for studying the microstructure, morphology and function of white matter fibers at the cellular and molecular levels. It studies the microstructure changes of white matter fibers in the brain regions of interest *in vivo* by measuring the diffusion of water (Raikes et al., 2018). The integrity and connectivity of white matter fiber is an important basis for the normal function of the brain (Lin et al., 2022). FA is the ratio of the fractional anisotropy to the total value of the diffusion tensor, which reflects the integrity of the cell membrane and myelin sheath and is sensitive to the orientation and consistency of the fiber tracts. MD is the average value of diffusion amplitude in all directions in a DTI imaging voxel, which represents the size or degree of diffusion of water molecules in a voxel and reflects the range of diffusion motion of water molecules per unit time. AD and RD are measures of the diffusion rate of water molecules along and perpendicular to the main diffusion direction in the brain region of interest, respectively. FA is often used in combination with other three diffusivity measures is to study the microstructure and function of brain region of interest (Walker et al., 2021). In this study, we investigated the microstructural changes in the MS and hippocampal CA1 regions based on the region of interest analysis method with the commonly used indicators of DTI include FA, MD, AD, and RD.

In this study, based on DTI white matter microstructure analysis, post-operative SF damaged the integrity of white matter fibers in the hippocampal CA1 region. The decrease of FA was highly sensitive to the detection of white matter microstructure damage in the CA1 region of the hippocampus, and the increase of RD and the decrease of AD indicated demyelination and axonal degeneration, respectively. However, we did not detect the change of hippocampal volume after post-operative 24-h SF. Although some studies have shown that sleep deprivation damages hippocampal structure and reduces hippocampal volume (Noorafshan et al., 2017), which in turn induces POCD (Wang et al., 2021), there is still a lack of credible high-quality evidence that short-term sleep problems induce significant changes in hippocampal volume. White matter fiber bundle injury in CA1 region is the structural basis of SF-induced POCD after surgery. Our data further strengthen the case that recurrent sleep fragmentation leads to structural and functional abnormalities in white matter associated with cognitive function.

Sleep fragmentation is related to the increased risk of various adverse health conditions in the elderly, mainly in terms of cognitive function (Kaneshwaran et al., 2019; Stewart and Arora, 2022). However, the effect of perioperative sleep fragmentation on POCD remains unclear. One study found that preoperative 24-h sleep fragmentation aggravated POCD in aged mice. However, another study suggested that preoperative and postoperative 24-h sleep fragmentation did not induce POCD in the adult mouse model (Vacas et al., 2017). Our behavioral tests showed that aged mice with postoperative SF were damaged in the Y maze and novel object recognition. Here, it should be noted that age itself is an independent risk factor for POCD. Studies have shown that in the aging period, the brain metabolism of mice is prone to disorder, the function of white matter fiber tracts decreased (Raikes et al., 2018). After anesthesia and surgery, 24-h sleep fragmentation treatment can significantly change the neuro-metabolism and white matter microstructure in the sleep and cognitive brain areas which also forms the pathological basis of POCD induced by SF in aged mice models.

This study expands our understanding of brain metabolism and white matter function in post-operative sleep fragmentation in the elderly, but has several limitations. Fist, 1H-MRS does not distinguish between extracellular and intracellular metabolite measurements and does not directly assess neurotransmitter transmission (Ding and Lanfermann, 2015), despite the use of a high-end 9.4T small animal MRI apparatus. Second, some neurometabolic products do not fully describe their physiological functions. In particular, the function of tCho and mI is to indirectly reflect the range of cholinergic neurons and glial cells, lacking specific molecular biological verification and supplement. Third, the MRI data we generated represent only one time point after post-operative sleep fragmentation. It is not known whether such drastic changes in brain metabolism and white matter fiber integrity over time also occur in the brain after behavioral tests. Fourth, the sample size of all our experiments is relatively small, and the corresponding molecular biology has not been implemented. Fifth, we cannot directly determine the relationship between the metabolic changes, microstructure changes, and POCD of these different neurotransmitters in the MS and hippocampal CA1 regions. Despite that, the current findings are sufficient to clarify the changes of neurotransmitter metabolism disorder and white matter fiber integrity damage in MS and CA1, the key brain regions of sleep and cognition in aged mice models, induced by postoperative sleep fragmentation.

## 5. Conclusion

We investigated the metabolites and structures of medial septum and hippocampal CA1 in aged mice models using 1H-MRS/DTI and found postoperative SF could lead to abnormal enhancement of glutamatergic, GABAergic and cholinergic metabolic signals in medial septum-hippocampal CA1, impairment of neuronal and white matter fiber integrity in hippocampal CA1 region, and postoperative cognitive dysfunction. These new findings provide a unique imaging reference for further studies to elucidate the neuro-metabolism and brain structure mechanism of post-operative sleep disturbance.

## Data availability statement

The original contributions presented in this study are included in the article, further inquiries can be directed to the corresponding authors.

## Ethics statement

This animal study was reviewed and approved by the Experimental Animal Welfare Ethics Committee of Tianjin Medical University General Hospital.

## Author contributions

YuL, KZ, LZ, and YoY designed the research. YiL, KZ, MS, JY, and JF conducted the research. YuL and LZ performed the statistical analysis and wrote the manuscript. YiL, YaY, and KX reviewed the manuscript. LZ and YoY supervised the research, revised the manuscript, and granted the funds. All authors contributed to the article and approved the submitted version.

## Footnotes

1
http://dsi-studio.labsolver.org/
